# The Protective Effect of Mulberry Leaf Flavonoids on High-Carbohydrate-Induced Liver Oxidative Stress, Inflammatory Response and Intestinal Microbiota Disturbance in *Monopterus albus*

**DOI:** 10.3390/antiox11050976

**Published:** 2022-05-16

**Authors:** Yong Shi, Lei Zhong, Yuding Fan, Junzhi Zhang, Huan Zhong, Xiang Liu, Chuang Shao, Yi Hu

**Affiliations:** 1 Hunan Research Center of Engineering Technology for Utilization of Distinctive Aquatic Resource, Hunan Agricultural University, Changsha 410128, China; shiyong@stu.hunau.edu.cn (Y.S.); zhonglei@hunau.edu.cn (L.Z.); zhjun123@hunau.edu.cn (J.Z.); zhonghuan@hunau.edu.cn (H.Z.); dreaml@stu.hunau.edu.cn (X.L.); shaoc@stu.hunau.edu.cn (C.S.); 2 Yangtze River Fisheries Research Institute, Chinese Academy of Fishery Sciences, Wuhan 430223, China; fanyd@yfi.ac.cn

**Keywords:** rice field eel, plant extract, immunity, antioxidant, intestinal health

## Abstract

An 8-week feeding trial with high-carbohydrate- and 100, 200 and 300 mg/kg mulberry leaf flavonoids (MLF)-supplemented diets (HCF1, HCF2 and HCF3, respectively) was conducted to evaluate the protective effect of MLF on oxidized high-carbohydrate-induced glucose metabolism disorder, liver oxidative damage and intestinal microbiota disturbance in *Monopterus albus*. The results showed that HC diets had significant negative effects on growth, glucose metabolism, liver antioxidant and immunity, as well as intestinal microbiota, in comparison to CON diets. However, WGR and SR in the HCF3 group dramatically increased compared to the HC group. With the increase of MLF in the HC diet, the activities of glycolysis and antioxidant enzymes in the liver tended to increase, while the changes of gluconeogenesis-related enzyme activities showed the opposite trend and significantly changed in the HCF3 group. Additionally, MLF supplementation dramatically increased the mRNA expression involved in glycolysis, antioxidative enzymes and anti-inflammatory cytokines in comparison with the HC group. Furthermore, gluconeogenesis and pro-inflammatory cytokine genes’ expression dramatically decreased. Furthermore, the proportion of *Clostridium* and *Rhodobacter* in the HC group dramatically declined, and the proportion of *Lactococcus* dramatically increased, compared to the HC group. In addition, 300 mg/kg MLF supplementation significantly improved the species composition and homeostasis of intestinal microbiota. These results indicate that MLF can alleviate the negative effects of low growth performance, glucose metabolism disorder, liver oxidative damage and intestinal microbiota disturbance caused by HC diets, and the relief of MLF is dose-related.

## 1. Introduction

With the decrease of fishmeal output and the increase in price, the production of a cost-effective diet (cost and effective) has attracted much attention [1]. At present, the mainstream strategy is to achieve a protein-saving effect in diets by supplementing carbohydrate and lipid. Compared with lipid sources, carbohydrates have a price advantage. Studies have shown that, by reducing the protein content in diets and filling it with energy substances (such as carbohydrates), the nitrogen excretion of aquatic animals can be reduced, thereby reducing water environmental pollution and reducing the cost of breeding [2,3].

Compared to terrestrial animals, aquatic animals have poorer utilization of carbohydrates [4]. Further studies have confirmed that carnivorous fish have weaker blood sugar clearance and glucose absorption capacity and thus, lower starch utilization capacity. Therefore, carnivorous fish have the lowest utilization of sugar compared with other fish [5]. Studies have shown that adding an appropriate carbohydrate into the diet can not only improve the growth performance but also antioxidant capacity in brook trout (*Salvelinus fontinalis*) [6]. However, when fish are fed excessive carbohydrate diets, it can lead to reduced growth performance, a low survival rate, physiological metabolism disorder and inflammatory response [7]. For example, when largemouth bass (*Micropterus salmoides*) and turbot (*Scophthalmus maximus* L.) were fed high-carbohydrate diets, there were negative effects such as reduced growth and disturbance of glucose metabolism [8,9]. In addition, high-carbohydrate diets induced high expression of proinflammatory factors in the head kidney of largemouth bass [10] and inhibited antioxidant enzyme activities (including CAT, SOD and GPx) in large largemouth bass [11]. Chinese perch (*Siniperca chuatsi*) fed with high-carbohydrate diets had disturbed intestinal microbiota [12]. Based on this, numerous studies have shown that feed additives are potential means to alleviate glucose metabolism disorders, oxidative stress and inflammation in fish. In particular, plant extracts have become a research hotspot as feed additives due to their various biological activities.

Mulberry (*Morus alba* L.) leaf is considered an excellent Chinese herbal medicine and contains many natural active substances, including flavonoids, polyhydroxyalkaloids, iminosugars, stilbenes, chlorogenic acids and benzofurans [13,14]. Therefore, mulberry leaf extract has attracted great attention in medicine. In clinical medicine, mulberry leaf extract has a variety of pharmacological effects, such as antibacterial, anti-inflammatory, antioxidant, anticancer and hypolipidemic effects, as well as promoting lipid metabolism, hypoglycemia and so on [13,15]. Similarly, dietary mulberry leaf extract supplementation alleviated high-fat-diet-induced lipid deposition, oxidative damage and inflammation in mice [16]; regulated intestinal microbiota homeostasis in pigs [17]; and improved growth, nutrient digestibility and meat quality in piglet and chicken broilers [18,19]. At present, mulberry leaf extract is also used as a high-quality additive in aquatic diets. Studies have reported that dietary mulberry leaf extract or leaves can improve growth performance and intestinal microbiota homeostasis in crucian carp (*Carassius carassius*) [20] and enhance antioxidant capacity and immune function in largemouth bass [21].

Flavonoids are one of the main functional components of mulberry leaves, accounting for about 1–3% of the dry matter of mulberry leaves. Mulberry leaf flavonoids enhanced glucose metabolism and mitochondrial function through activating AMPK-PGC1α signaling pathways, alleviated liver and kidney injury and improved antioxidant capacity in type 2 diabetes [22,23]. In addition, Zhong et al. found that mulberry leaf flavonoids can alleviate high-fat-induced lipid dysmetabolism induced through intestinal microbiota in mice [24]. In aquatic animals, studies have indicated that dietary mulberry leaf flavonoids can improve growth performance and resistance to nitrite exposure in tilapia (*Oreochromis niloticus*) [25], promote intestinal mucosal morphology and regulate the diversity of intestinal microbiota in *Litopenaeus vannamei* [26]. Therefore, the potential benefits of mulberry leaf flavonoids as additives in high-carbohydrate diets deserve further study.

Rice field eels, a carnivorous fish with high nutritional value, are widely cultivated in China. Studies have shown that the carbohydrate content in the optimal diet for rice field eel growth is 24–33% [27]. In this study, rice field eel was used as the research object to test the hypothesis that mulberry leaf flavonoids can ameliorate the negative effects caused by high-carbohydrate diets. Our objective was to evaluate the growth performance, glucose metabolism, liver antioxidant capacity and intestinal microbiota of rice field eel to determine whether mulberry leaf flavonoids have beneficial effects on fish fed high-carbohydrate diets and to provide a basis for the application of mulberry leaf flavonoids in high-carbohydrate diets.

## 2. Materials and Methods

### 2.1. Animals, Experimental Design, Diet Preparation and Culture Conditions

Rice field eel juveniles were obtained from Xihu Farm, Changde, China. The acclimatization method was consistent with our previous research [28]. After this, 900 healthy rice field eel juveniles (initial weight 14.97 ± 0.06 g) randomly allocated to five diets (three cages per dietary treatment, 60 fish per cage): CON diets (20% corn starch); HC diets (40% corn starch); or HC diets supplemented with 100, 200 and 300 mg/kg mulberry leaf flavonoids (HCF1, HCF2 and HFC3, respectively) (Table 1). The mulberry leaf flavonoids (purity > 85%, total flavonoids) were provided by Sericulture and Agricultural Products Processing Institute of Guangdong Academy of Agricultural Sciences. Diet preparation was the same as in a previous study [28]. The breeding experiment lasted for 8 weeks, and the feeding amount was 3–5% of body weight once a day (17:00–18:30 h). Water conditions including pH (7.3 ± 0.2), temperature (28.6 ± 2.2 °C), dissolved oxygen (6.3 ± 0.4 mg/L) and ammonia nitrogen (0.12 ± 0.04 mg/L) were measured. 

### 2.2. Sample Collection

After the feeding trial, fish were collected after 24 h of fasting. All fish were euthanized (MS-222 at 100 μg/mL). Serum collection was consistent with previous studies [29]. Liver samples (3 fish per cage) were collected and stored at −80 °C. The intestinal contents of three fish per cage were also collected and stored at −80 °C until further analysis of intestinal microbiota.

### 2.3. Chemical Analysis

The measurement methods of crude lipid, crude protein and ash in diets were consistent with previous studies [29].

### 2.4. Serum Biochemical Indices Analysis

Glucose (GLU), triacylglycerol (TG), total cholesterol (TC), high-density lipoprotein cholesterol (HDL-C) and low-density lipoprotein cholesterol (LDL-C) were enzymolyzed, respectively, to produce H_2_O_2_, which could generate red benzoquinone imine under the catalytic action of peroxidase. The products had characteristic absorption peaks at 505, 510, 510, 546 and 546 nm, respectively, and the contents of GLU, TG, TC, HDL-C and LDL-C could be determined by the change in the absorbency value.

Alanine aminotransferase (ALT) and aspartate aminotransferase (AST), respectively, catalyze the transamination reaction to produce pyruvate, which can react with 2,4-dinitrophenylhydrazine to produce 2,4-dinitrophenylhydrazone, which is brownish red under alkaline conditions. The products had characteristic absorption peaks at 510 nm, and the activities of ALT and AST could be determined by the change in the absorbency value.

### 2.5. Liver Glucose Metabolism and Antioxidant Indices Analysis

The levels of reactive oxygen species (ROS), pyruvate kinase (PK), glucokinase (GK), glucose-6-phosphatase (G-6-Pase), phosphoenolpyruvate carboxykinase (PEPCK), fructose-1.6-bisphosphatase (FBPase) and phosphofructokinase (PFK) in each sample were measured using Elisa kits [30]. The detection method for catalase (CAT), glutathione (GSH), and superoxide dismutase (SOD) levels in the liver were the same as in the previous study [29]. The glutathione peroxidase (GPx) activity in the liver was spectrophotometrically measured at 412 nm.

### 2.6. Total RNA Extraction, Reverse Transcription and Real-Time PCR

The procedures of total RNA extraction, reverse transcription and qRT-PCR were performed, using methods previously used in our laboratory [31]. The amplification efficiency of all genes was approximately equal and ranged from 0.95 to 1.10. The relative expression level of genes was calculated by the formula E = 2^−ΔΔCT^ [32]. Gene primer sequences were designed by Primer Premier 5.0 (Table 2). The internal reference gene was ribosomal protein L-17 (*rpl-17*) [33].

### 2.7. Intestinal Microbiota Analysis

In this study, the Illumina platform was used for the paired-end sequencing of community DNA fragments. The DADA2 method was used for sequence denoising [34]. Vsearch (V2.13.4_linux_x86_64) was used for clustering. Sequence data analyses were mainly performed using QIIME2 and R packages (v3.2.0). Alpha diversity indices, such as Chao1, observed species, Shannon diversity index, Simpson index and Good’s coverage, were calculated using the table in QIIME2 and visualized as box plots. Species analysis at the phylum and genus level was the same as in our previous study [31].

### 2.8. Statistical Analysis

All data (mean ± SE) were analyzed by one-way ANOVA. After testing for homogeneity of variances, Duncan multiple comparisons were performed. *p* values less than 0.05 were considered significant differences. All graphs were drawn using Graphpad software (San Diego, CA, USA). SPSS 24.0 software (New York, NY, USA) was used for all statistical analysis.

## 3. Results

### 3.1. Growth and Morphology Parameters

No significant differences were showed in final weight, VSI, CF or FCR (*p* > 0.05) (Table 3). The rice field eel fed HC diets had significantly lower WGR and SR than those fed CON and HCF3 diets (*p* < 0.05). Dietary 300 mg/kg mulberry leaf flavonoids significantly decreased HSI compared to the HC group (*p* < 0.05).

### 3.2. Serum Biochemical Indices

The rice field eel fed HC diets had significantly higher AST, ALT, TG, TC, GLU and LDL-C concentrations than those fed CON diets (*p* < 0.05) (Table 4). However, feeding the rice field eel mulberry leaf flavonoids (100, 200 and 300 mg/kg) markedly decreased serum AST, ALT, TG, TC, GLU and LDL-C concentrations (*p* < 0.05). Dietary 200 and 300 mg/kg mulberry leaf flavonoids significantly increased HDL-C concentration compared to the HC group (*p* < 0.05).

### 3.3. Carbohydrate Metabolism Enzyme Activity

**Figure 1:**
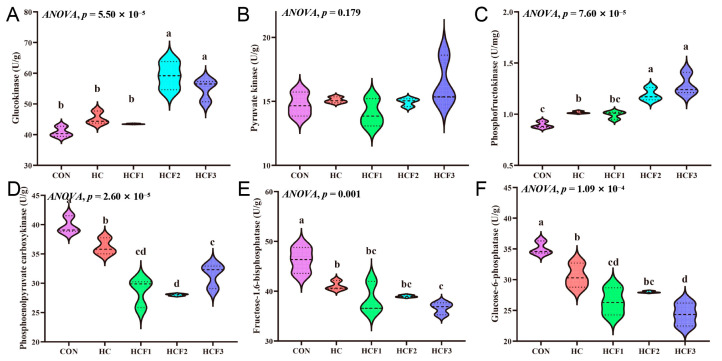
Influences of dietary mulberry leaf flavonoids on carbohydrate metabolism enzymes activities in liver in rice field eel fed on a high-carbohydrate diet. (**A**) Glucokinase, GK; (**B**) Pyruvate kinase, PK; (**C**) Phosphofructokinase, PFK; (**D**) Phoenolpyruvate carboxykinase, PEPCK; (**E**) Fructose-1.6-bisphosphatase, FBPase; (**F**) Glucose-6-phosphatase, G-6-Pase. Different letters show a significant difference (*p* < 0.05).

No significant differences were shown in liver PK concentration (*p* > 0.05) (Figure 1). Compared with the CON and HC groups, PEPCK activity in the HCF1, HCF2 and HCF3 groups was significantly decreased (*p* < 0.05). HC diets significantly increased liver PFK activity and decreased liver PEPCK, FBPase and G-6-Pase concentrations compared to the CON group (*p* < 0.05). However, feeding the rice field eel mulberry leaf flavonoids (100, 200 and 300 mg/kg) markedly decreased liver PEPCK activity (*p* < 0.05). Dietary 200 and 300 mg/kg mulberry leaf flavonoids also significantly increased GK and PFK concentrations compared to the HC group (*p* < 0.05). FBPase and G-6-Pase concentrations in the HCF3 treatment were markedly low than those in the HC group (*p* < 0.05).

### 3.4. Genes Expression of Carbohydrate Metabolism

**Figure 2:**
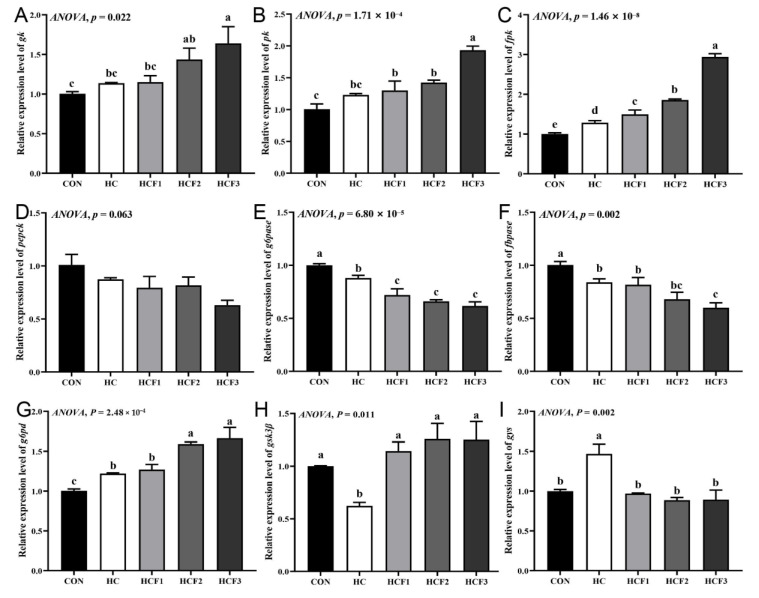
Influences of dietary mulberry leaf flavonoids on carbohydrate metabolism relative genes expression in liver in rice field eel fed on a high-carbohydrate diet. (**A**) *gk*; (**B**) *pk*; (**C**) *fpk*; (**D**) *pepck*; (**E**) *g6pase*; (**F**) *fbpase*; (**G**) *g6pd*; (**H**) *gsk3β*; (**I**) *gys*. Different letters show significant difference (*p* < 0.05).

No significant differences were shown in the mRNA expression of liver *pepck* (*p* > 0.05) (Figure 2). HC diets significantly upregulated the mRNA expression of *fpk*, *g6pd* and *gys* compared to the CON group (*p* < 0.05), with downregulated gene expression of *g6pase*, *fbpase*, and *gsk3β* (*p* < 0.05). Furthermore, 100, 200 and 300 mg/kg mulberry leaf flavonoid supplementation in HC-fed rice rield eel significantly downregulated the mRNA expression of *g6pase* and *gys*, as well as upregulated the mRNA expression of *fpk* and *gsk3β* (*p* < 0.05). The HCF3 group significantly reduced the mRNA expression of *fbpase* and increased the mRNA expression of *gk*, *pk* and *g6pd* compared to the HC group (*p* < 0.05).

### 3.5. Antioxidant Indices in the Liver

**Figure 3:**
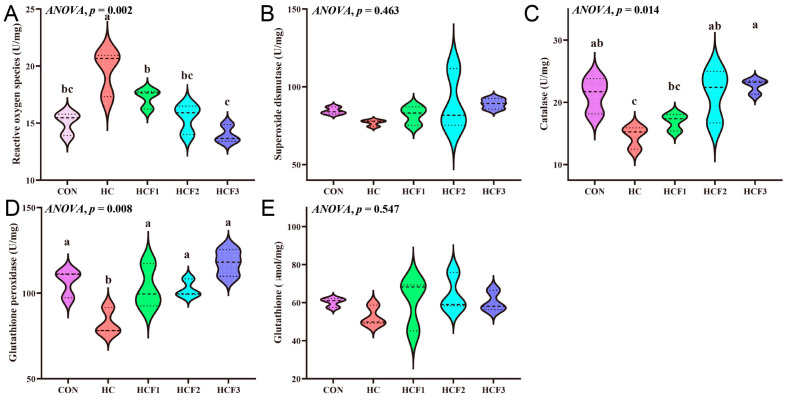
Influences of dietary mulberry leaf flavonoids on antioxidant enzymes activities in the liver of rice field eel fed on a high-carbohydrate diet. (**A**) Reactive oxygen species, ROS; (**B**) Superoxide dismutase, SOD; (**C**) catalase, CAT; (**D**) Glutathione peroxidase, GPx; (**E**) Glutathione, GSH. Different letters show a significant difference (*p* < 0.05).

No significant differences were showed in liver SOD and GSH concentrations (*p* > 0.05) (Figure 3). The rice field eel fed HC diets had significantly higher ROS content and lower CAT and GPx activities than those fed CON diets. However, feeding the rice field eel mulberry leaf flavonoids (100, 200 and 300 mg/kg) markedly decreased liver ROS content and increased liver GPx activity (*p* < 0.05), which was similar to results from the CON group (*p* > 0.05). Dietary 200 and 300 mg/kg mulberry leaf flavonoids also significantly increased CAT activity compared to the HC group (*p* < 0.05).

### 3.6. Genes Expression of Liver Antioxidant

**Figure 4:**
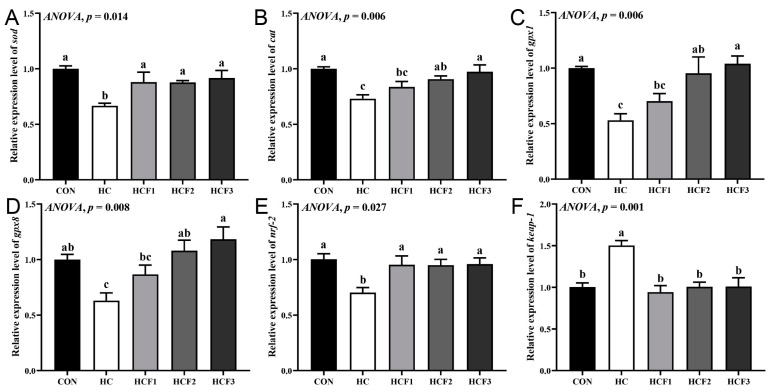
Influences of dietary mulberry leaf flavonoids on antioxidant-related genes’ expression in the liver of rice field eels fed on a high-carbohydrate diet. (**A**) *sod*; (**B**) *cat*; (**C**) *gpx1*; (**D**) *gpx8*; (**E**) *nrf-2*; (**F**) *keap1*. Different letters show a significant difference (*p* < 0.05).

The rice field eel fed HC diets had significantly higher mRNA expression of *keap1* and lower mRNA expression of *gpx1*, *gpx8*, *cat*, *sod* and *nrf-2* than those fed CON diets (*p* < 0.05) (Figure 4). However, feeding the rice field eel mulberry leaf flavonoids (100, 200 and 300 mg/kg) markedly decreased liver *keap1* mRNA expression and increased *sod* and *nrf-2* mRNA expression (*p* < 0.05), which was similar to the CON group (*p* > 0.05). Dietary 200 and 300 mg/kg mulberry leaf flavonoids also significantly increased *gpx1*, *gpx8* and *cat* mRNA expression compared to the HC group (*p* < 0.05). 

### 3.7. Gene Expression Related to the Liver Damage

**Figure 5:**
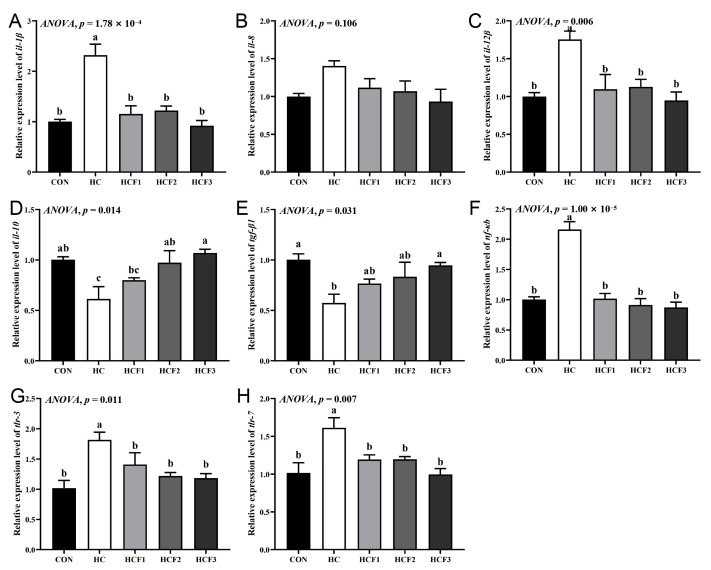
Influences of dietary mulberry leaf flavonoids on inflammation-related genes’ expression in the liver of rice field eel fed on a high-carbohydrate diet. (**A**) *il-1β*; (**B**) *il-8*; (**C**) *il-12β*; (**D**) *il-10*; (**E**) *tgf-β1*; (**F**) *nf-κb*; (**G**) *tlr-3*; (**H**) *tlr-7*. Different letters show a significant difference (*p* < 0.05).

The rice field eel fed HC diets had significantly higher mRNA expression of *il-1β*, *il-12β*, *tlr-3*, *tlr-7* and *nf-κb* and lower mRNA expression of *il-10* and *tgf-β1* than those fed CON diets (*p* < 0.05) (Figure 5). The supplementation of 100–300 mg/kg mulberry leaf flavonoids remarkably downregulated *il-1β*, *il-12β*, *tlr-3*, *tlr-7* and *nf-κb* mRNA expression compared to the HC group (*p* < 0.05). *il-10* mRNA expression in the HCF2 and HCF3 groups and *tgf-β1* mRNA expression in the HCF3 group were remarkably downregulated compared to the HC diet (*p* < 0.05). With the increase in the mulberry leaf flavonoid supplemental level, the mRNA expression of *tgf-β1* and *il-10* showed an increasing trend, while the mRNA expression of *il-1β*, *tlr-3* and *tlr-7* showed a decreasing trend.

**Figure 6:**
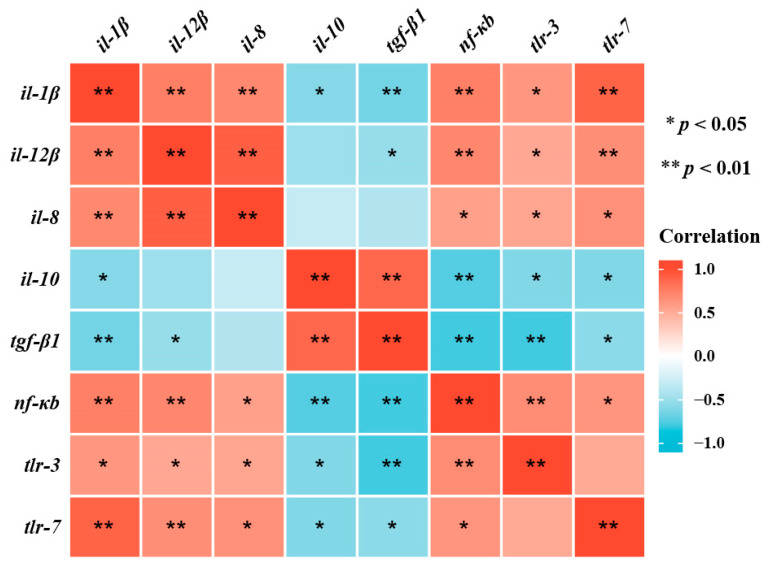
Correlative analysis of liver-inflammation-related gene expression. * *p* < 0.05, ** *p* < 0.01.

Correlation analysis revealed that the mRNA transcription level of *nf-κb* was significantly (*p* < 0.05) positively correlated with that of the mRNA transcription levels of *il-10* and *tgf-β1* and negatively correlated with that of the mRNA transcription levels of *il-1β*, *il-8*, *il-12β*, *tlr-3* and *tlr-7* (Figure 6).

### 3.8. Intestinal Microbiota Analysis

#### 3.8.1. Diversity Analysis

**Figure 7:**
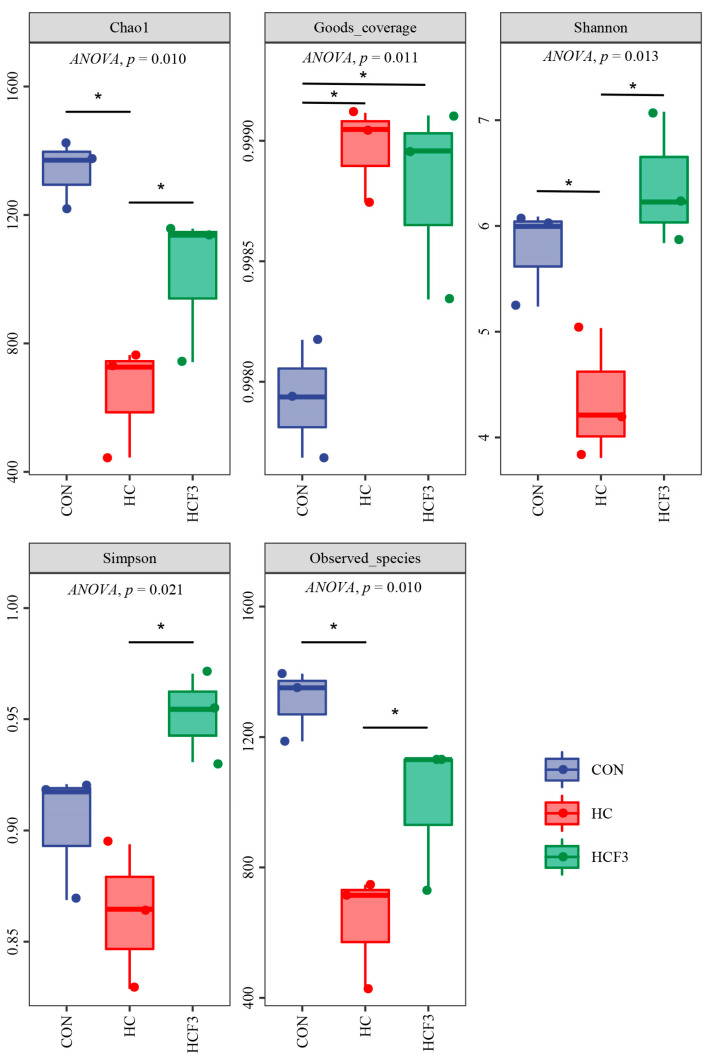
Influences of dietary mulberry leaf flavonoids on the alpha-diversity of intestinal microbiota in rice field eel fed a high-carbohydrate diet. * indicates a significant difference between groups (*p* < 0.05).

No significant differences were showed in the Simpson index (*p* > 0.05) (Figure 7). HC diets significantly decreased Chao1, Shannon and observed species indexes in comparison with the CON group (*p* < 0.05). A total of 300 mg/kg mulberry leaf flavonoid supplementation significantly increased the Chao1, Shannon and observed species indexes in comparison with the HC group (*p* < 0.05).

#### 3.8.2. Microbial Composition

**Figure 8:**
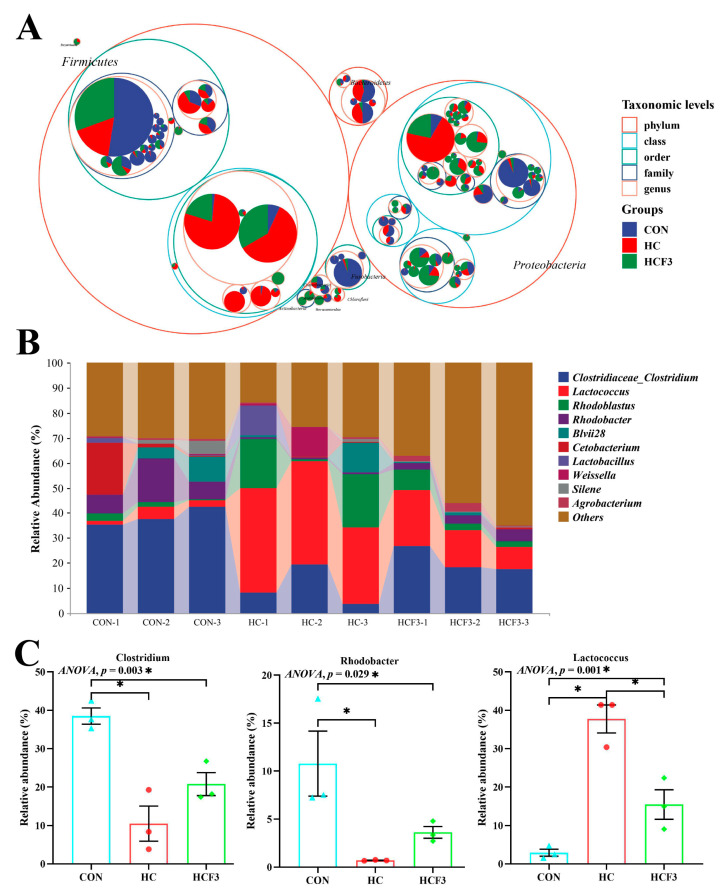
Effects of dietary mulberry leaf flavonoids on the intestinal microbial composition of rice field eel (*Monopterus albus*) fed high-carbohydrate diets. (**A**) Classification hierarchy tree diagram (The largest circle represents phylum level, and the decreasing circle represents class, order, family, genus and species in descending order). (**B**) Species abundance at the genus level (Top 10), (**C**) showing significant variations of the relative abundance of intestinal microbiota at the genus level. * indicates a significant difference between groups (*p* < 0.05).

The tree diagram shown that no significant differences were shown in dominant intestinal microbiota at the phylum level (*p* > 0.05), mainly including *Firmicutes* and Proteobacteria (Figure 8A). The rice field eel fed HC diets had significantly higher abundance of Lactococcus and lower abundance of *Clostridium* and *Rhodobacter* than those fed CON diets (*p* < 0.05) (Figure 8B,C). The supplementation of 300 mg/kg mulberry leaf flavonoids remarkably decreased the abundance of *Lactococcus* and increased the abundances of *Clostridium* and *Rhodobacter* in comparison with the HC group (*p* < 0.05), without reaching the CON group.

## 4. Discussion

The appropriate dietary carbohydrate content can improve the growth performance of kelabau (*Osteochilus melanopleurus*) [35]. However, HC diets significantly decreased the growth performance of *Lateolabrax japonicus* [36], cobia (*Rachycentron canadum* L.) [6], hybrid grouper [37] and largemouth bass [38]. The results of this study demonstrated that rice field eels fed a HC diet had a significantly reduced weight-gain rate. The main reason for this may be their low carbohydrate-utilization capacity; studies have shown that carnivorous fish are natural diabetic patients [5]. Functional additives are a common method for improving carbohydrate utilization in aquatic animals. The flavonoids of *Alnus glutinosa* can improve the growth performance of mutton sheep by promoting the secretion of growth hormone and insulin-like growth factor-1 in the serum [39]. Similarly, dietary mulberry leaf flavonoids improved the growth performance of tilapia [25]. Flavonoids from *Allium mongolicum Regel* have also been found to improve the growth performance in juvenile northern snakehead fish (*Channa argus*) [40]. This study also demonstrated that supplementation of diets with 300 mg/kg mulberry leaf flavonoids significantly improved the weight-gain rate of the HC-diet-fed rice field eel, which was similar to results from the CON group. This indicates that mulberry leaf flavonoids increased growth performance in rice field eels. 

High serum-glucose content has previously been found in largemouth bass and tilapia fed high-carbohydrate diets [38,41]. In this study, long-term feeding of HC diets significantly increased serum GLU content in rice field eels. However, liver tissue plays an important role in regulating blood glucose homeostasis [42]. Several important pathways are involved in this regulation, such as gluconeogenesis and glycolysis, in which G-6-Pase, FBPase and PEPCK activities are restriction enzymes in the gluconeogenesis pathway, and PFK, PK and GK are the limiting enzymes in the glycolysis pathway [43]. This study showed that rice field eel fed HC diets had significantly higher PFK and GK concentrations and lower FBPase, G-6-Pase and PEPCK concentrations than those fed CON diets. In addtion, the mRNA expression of *fpk*, *gk*, *fbpase*, *g6pase* and *pepck* were the same as their enzyme activity changes. Similar results were seen in blunt snout bream (*Megalobrama amblycephala*) [41] and largemouth bass [10].

Mulberry leaf is a Chinese medicinal supplement that is widely used to regulate blood glucose [44]. Flavonoids, one of the main active ingredients in mulberry leaves, have the potential to treat type II diabetes [23,45]. In this study, the addition of mulberry leaf flavonoids, especially at the higher level (300 mg/kg), remarkably downregulated the transcriptional levels of *fbpase* and *g6pase* and upregulated the transcriptional levels of *fpk*, *pk* and *gk* of HC-diet-fed fish, and the corresponding enzyme activity results were consistent with them. Some studies have reported that Gys plays a very important role in the final stage of catalyzing glycogen synthesis during carbohydrate metabolism, and *gys* mRNA expression is negatively correlated with *gsk3β* [46]. This study showed that compared to the CON diet, the HC diet induced significantly lower liver *gsk3β* mRNA expression and higher *gys* mRNA expression. However, the addition of mulberry leaf flavonoids to HC diets reversed this trend. Thus, these results indicate that mulberry leaf flavonoids reduced serum-glucose content by promoting glycolysis and inhibiting the gluconeogenesis pathway.

According to previous studies, excessive carbohydrate intake can cause severe physiological stress, such as oxidative stress and inflammation [47,48]. In this study, high-carbohydrate diets induced relatively high levels of serum ALT and AST activity; liver ROS content; and liver *il-1β*, *il-8* and *il-12β* mRNA expression levels compared to the CON group. In contrast, the hepatic activities of CAT, GPx and liver *il-10* and *tgf-β1* mRNA expression levels were relatively low. These findings indicate that long-term feeding of a HC diet causes oxidative stress and induces inflammation in rice field eels. The above conclusions can be supported by the following reasons: first, studies have shown that a decrease in antioxidant enzyme (such as GPx, CAT and SOD) activity and an increase in ROS content are the symbolic signals of oxidative damage in fish [49,50,51]; Second, evidence of high-carbohydrate diet-induced liver inflammatory damage was supported by the increase in serum AST and ALT activities, which is an important marker of liver damage [52], as well as *il-1β* and *il-8*, which are the main pro-inflammatory factors [53].

The alleviation of the HC-diet-induced oxidative stress and inflammatory response imperative is improved by the application of carbohydrates in aquatic feed. The flavonoids in mulberry leaves are natural antioxidants, which can significantly increase SOD, CAT and CPx activities and significantly reduce MDA content, thereby improving antioxidant capacity and reducing oxidative stress [54]. Moreover, flavonoids are known to be effective immune enhancers [55]. In this study, the addition of mulberry leaf flavonoids, especially at the higher level (300 mg/kg), significantly increased hepatic CAT and GPx activities, as well as the liver *il-10* and *tgf-β1* mRNA expression levels of HC-diet-fed fish, while decreasing serum AST and ALT activities; liver MDA content; and liver *il-1β*, *il-8* and *il-12β* mRNA expression levels. These findings indicate that dietary mulberry leaf flavonoid supplementation enhances antioxidant capacity and alleviates inflammation in the liver of rice field eels fed high-carbohydrate diets. According to previous studies, dietary flavonoid supplementation can significantly enhance antioxidant ability (such as CAT, SOD and GPx) in farmed tilapia [25] and reduce significant the chromium-induced upregulation of pro-inflammatory factors (such as *tnf-α* and *il-1β*) in *Ctenopharyngodon idella* [56]. Furthermore, dietary flavonoids from *Allium mongolicum Regel* can reduce Se-accumulation in organs, decrease oxidative stress, increase immune response and regulate immune-related signaling molecules following Se exposure in *Channa argus* [57]. In the same way, the excellent antioxidant properties of mulberry leaf flavonoids are derived from their ability to directly scavenge ROS by providing a hydrogen atom or single-electron transfer and immediately chelate free radicals [58]. In this study, dietary mulberry leaf flavonoid supplementation significantly decreased ROS content. This further supports the antioxidant capacity of mulberry leaf flavonoids.

Antioxidant capacity and inflammatory responses in animals are regulated by the Nrf2/Keap1 and NF-κB/TLRs signaling pathways, respectively [31,59]. A previous study showed that high-carbohydrate diets induce oxidative damage and inflammatory responses by influencing the Nrf2 and NF-κB signaling pathways in blunt snout bream [60]. *keap1* inhibits the expression of antioxidant genes by inhibiting the nuclear translocation of *nrf2* [61]. This study showed that HC diets remarkably upregulated the transcriptional levels of *keap1*, *nf-κb*, *tlr-3* and *tlr-7* in the liver, whereas the transcriptional level of *nrf2* was downregulated. These findings indicate that high-carbohydrate diets lead to oxidative stress and inflammation by activating the NF-κB signaling pathway and inhibiting the Nrf2 signaling pathway. Subsequently, the addition of mulberry leaf flavonoids to HC diets remarkably downregulated the transcriptional levels of *keap1*, *nf-κb*, *tlr-3* and *tlr-7* in the liver, whereas the transcriptional level of *nrf2* was upregulated. Furthermore, correlation analyses showed that the transcriptional level of *nf-κb* was negatively correlated with the mRNA expression levels of *il-1β*, *il-8*, *il-12β*, *tlr-3* and *tlr-7*, and positively correlated with the mRNA expression levels of *il-10* and *tgf-β1*. This suggests that mulberry leaf flavonoids inhibited the NF-κB signaling pathway to protect high-carbohydrate-induced inflammatory response in rice field eels. Similar studies have been conducted on flavonoids extracted from other plants. For example, flavonoids extracted from *Barnebydendron riedelii* can enhance antioxidant capacity by inhibiting the Nrf2 signaling pathway [62], and flavonoids extracted from cocoa can alleviate the inflammatory response by inhibiting the NF-κB signaling pathway [63]. These results indicate that plant flavonoids could effectively enhance the antioxidant capacity and inhibit inflammation via the Nrf2/Keap1 and NF-κB/TLRs signaling pathways in rice field eels fed HC diets.

Numerous studies have shown that the intestinal microbiota plays a beneficial role for the host in diabetes, oxidative damage and inflammatory responses [64,65]. Alpha-diversity (Chao1, Shannon and observed species index) is an important indicator for judging the species richness of intestinal microbiota. In this study, the rice field eel fed HC diets had significantly lower Chao1, Shannon and observed species indices than those fed CON diets, and similar results were found in Nile tilapia and Chinese perch [12,41]. These results indicate that fish fed HC diets for a long time showed reduced diversity of intestinal microbiota. 

In addition, we also found that HC-diet feeding remarkably increased the abundance of *Lactococcus* and reduced the abundance of *Clostridium* and *Rhodobacter* in rice field eels. However, *Clostridium* is able to promote the production of butyrate, a short-chain fatty acid that scavenges oxygen and free radicals and enhances host antioxidant immunity [66,67,68]. *Lactococcus*, a globular gram-positive anaerobe, causes adverse host reactions, including inflammatory responses and low growth rates [69,70]. *Rhodobacter* gram-negative bacteria have been used to produce terpenes, which have various biological functions [71]. However, in this study, HC diets resulted in a significant decrease in *Clostridium* and *Rhodobacter*, which may reduce their metabolites and lead to physiological disorders. However, the specific metabolites involved require further study. These results may indicate that oxidative stress and inflammatory responses induced by high-carbohydrate diets are closely related to intestinal microbiota homeostasis.

Flavonoids interact with intestinal microbiota, which can participate in the transforming flavonoids in vivo. For example, *Clostridium* participates in the metabolism of flavonoids in vivo and has a stronger physiological function [26]. Flavonoids also support the growth of normal bacteria, inhibit the proliferation of harmful bacteria and regulate the intestinal microecological balance [72]. Previous research has confirmed that *Cyclocarya paliurus* flavonoids can positively regulate the composition and function of intestinal microbiota disturbed by host circadian disturbances, thus improving the intestinal microecological balance in mice [73]. The present study also showed that 300 mg/kg mulberry leaf flavonoid supplementation in HC diets caused no significant difference in Chao1, Shannon and observed species compared to that in the CON group. Furthermore, the supplementation of 300 mg/kg mulberry leaf flavonoids remarkably decreased the abundance of *Lactococcus* and increased the abundance of *Clostridium* and *Rhodobacter* in comparison with the HC group. In pre-weaned calves, dietary mulberry leaf flavonoids can reduce the number of diarrhea days after *Escherichia coli* K99 infection, effectively regulate the intestinal microbiota and improve intestinal health [74]. These findings indicate that mulberry leaf flavonoids could restore the intestinal microbiota dysbiosis induced by HC diets. Furthermore, the enhancement of the antioxidant capacity and anti-inflammatory capacity of mulberry leaf flavonoids is closely related to the regulation of the intestinal microbiota of rice field eels. However, the specific regulatory mechanism requires further study.

## 5. Conclusions

We have shown that HC-diet feeding had an adverse effect on growth, carbohydrate metabolism, liver antioxidant, immune capacity and intestinal microbiota. However, dietary mulberry leaf flavonoid supplementation increased growth performance in rice field eels. In addition, mulberry leaf flavonoids increased carbohydrate metabolism, enhanced liver immune and antioxidant capacity and regulated intestinal microbiota homeostasis in eels fed HC diets. The optimal addition amount of mulberry leaf flavonoids was 300 mg/kg.

## Figures and Tables

**Table 1:** Formulation and proximate composition of the experimental diets (%, dry matter).

Ingredients	CON	HC	HCF1	HCF2	HCF3
Fish meal ^a^	40.00	40.00	40.00	40.00	40.00
Wheat gluten ^a^	14.46	14.46	14.46	14.46	14.46
Corn starch ^a^	20.00	40.00	40.00	40.00	40.00
Microcrystalline cellulose ^a^	20.50	0.50	0.49	0.48	0.47
Fish oil ^a^	2.00	2.00	2.00	2.00	2.00
Choline ^a^	0.50	0.50	0.50	0.50	0.50
Premix ^b^	1.00	1.00	1.00	1.00	1.00
Ca(H_2_PO_4_)_2_ ^a^	1.50	1.50	1.50	1.50	1.50
Antioxidants ^a^	0.01	0.01	0.01	0.01	0.01
Mold inhibitor ^a^	0.03	0.03	0.03	0.03	0.03
Mulberry leaf flavonoids ^c^	0.00	0.00	0.01	0.02	0.03
Proximate composition (%)					
Crude protein	43.86	43.72	44.04	43.87	43.79
Crude lipid	5.86	5.93	5.82	5.79	5.87
Ash	10.85	11.03	10.94	10.72	10.83

^a^ Provided by Hunan Aohua Agriculture and Animal Husbandry Technology Co. Ltd. (Changde, China). ^b^ Provided by MGO Ter Bio-Tech (Qingdao, China). Composition consistent with previous studies [28]. ^c^ Provided by Sericulture and Agricultural Products Processing Institute of Guangdong Academy of Agricultural Sciences.

**Table 2:** Primers used for mRNA quantitative real-time PCR.

Gene	Forward Sequences (5′→3′)	Reverse Sequences (5′→3′)	Accession No.
*gk*	AAGCCATCGTATCCCACC	GGGTCCCAGTCCATAGTGT	XM_020620531.1
*pk*	CCGCCAAGGGACTGTTT	CCACTGGTGGTAAGGACTATG	XM_020617665.1
*fpk*	AGCATAGGAGCAGACACCG	CACAGAATCCACCCATAGTC	XM_020594803.1
*g6pd*	CCACCCACTGTCTACCA	GGCTCTGCACCATTTCT	XM_020610610.1
*pepck*	CTGTGACGGCTCTGACG	ATACATGGTGCGACCTTTC	XM_020621224.1
*g6pase*	GGTATGAGGGTCTGTTTAGC	GACAGCCACCCAGATGA	XM_020616553.1
*fbpase*	GCTGCGGTTGCTGTATG	TTCTTGGCGTGTTTATGG	XM_020585913.1
*gsk3β*	GGTGTTGTCTACCAGGCTAA	CAATGGTCCAACTTCCTCA	XM_020609458.1
*gys*	CGGCTGCCAGGTTTATT	GCCCAGGATGAGCGAGT	XM_020608327.1
*nrf2*	CTTCAGACAGCGGTGACAGG	GCCTCATTCAGTTGGTGCTT	XM_020596409.1
*keap1*	AGCCTGGGTGCGATACGA	CAAGAAATGACTTTGGTGGG	XM_020597068.1
*sod*	AGCTGGCTAAGTTCTCATTCAC	GCAGTAACATTGCCCAAGTCT	XM_020598413.1
*cat*	GTCCAAGTCTAAGGCATCTCC	CTCCTCTTCGTTCAGCACC	XM_020624985.1
*gpx1*	GTTCACCGCCAAACTCTT	TTCCCATTCACATCTACCTT	XM_020607739.1
*gpx8*	GTCCACTTACGGTGTTACCT	ATGGGCTCGTCAGTTCTC	XM_020593975.1
*il-1β*	GAGATGTGGAGCCCAAACTT	CTGCCTCTGACCTTCTGGACTT	KM113037.1
*il-8*	TACTGGTTCTGCTTACTGTCGC	CAAATCTTTTGCCCATCCCT	XM_020597077.1
*il-12β*	CAAGTCAGTTGCCAAAATCC	CCAAGCAGCTCAGGGTCT	XM_020594580.1
*il-10*	TTTGCCTGCCAAGTTATGAG	CATTTGGTGACATCGCTCTT	XM_020593225.1
*tgf-β1*	AACCCACTACCTCACTACCCG	GCCGAAGTTGGAAACCCT	XM_020605575.1
*nf-κb*	ACCCTACCGTGACACTAACCT	TGCCGTCTATCTTGTGGAAT	XM_020616319.1
*tlr-3*	TATTTAGAGCCATACAGGG	CACAATCAAGAACGCACA	XM_020614353.1
*tlr-7*	ATCCTCACGACTTCCCTC	TTTCTTTCATCACCCACT	XM_020596482.1
*rpl-17*	GTTGTAGCGACGGAAAGGGAC	GACTAAATCATGCAAGTCGAGGG	109952565

**Table 3:** Effects of dietary mulberry leaf flavonoids on the growth performance of *Monopterus albus* fed high-carbohydrate diets (mean ± S.E.).

	CON	HC	HCF1	HCF2	HCF3	*p*-Value
Initial weight (g)	14.96 ± 0.12	14.97 ± 0.24	15.11 ± 0.12	14.96 ± 0.02	15.01 ± 0.03	0.919
Final weight (g)	36.56 ± 0.94	33.08 ± 1.02	34.96 ± 0.60	35.58 ± 1.50	37.13 ± 0.70	0.112
WGR	144.26 ± 4.47 ^a^	120.92 ± 3.27 ^b^	131.41 ± 3.57 ^ab^	137.82 ± 9.71 ^ab^	147.37 ± 4.24 ^a^	0.046
SR	91.11 ± 2.78 ^a^	77.78 ± 3.38 ^b^	77.22 ± 2.00 ^b^	81.66 ± 3.33 ^ab^	88.33 ± 2.89 ^a^	0.023
FCR	1.39 ± 0.06	1.67 ± 0.07	1.52 ± 0.04	1.47 ± 0.10	1.36 ± 0.04	0.051
HSI	3.44 ± 0.15 ^ab^	3.92 ± 0.07 ^a^	3.63 ± 0.08 ^ab^	3.49 ± 0.13 ^ab^	3.32 ± 0.24 ^b^	0.048
VSI	15.73 ± 0.71	17.22 ± 0.81	18.00 ± 1.28	15.65 ± 1.88	17.68 ± 1.07	0.533
CF	9.00 ± 0.32	10.20 ± 0.49	9.80 ± 0.66	10.00 ± 0.32	10.00 ± 0.55	0.465

Note: In the same row values with different small letter superscripts mean significant difference (*p* < 0.05). Weight gain rate (WGR, %) = (final body weight − initial body weight)/initial body weight × 100; survival rate (SR, %) = final number of fish/initial number of fish × 100; feed-conversion rate (FCR) = total amount of the feed consumed/(final body weight − initial body weight); hepatosomatic index (HSI, %) = liver weight/whole body weight × 100; viserosomatic index (VSI, %) = visceral weight/whole body weight × 100; Condition factor (CF, g/cm^3^) = 100 × whole body weight/(body length)^3^.

**Table 4:** Effects of dietary mulberry leaf flavonoids on serum biochemical indices of rice field eel (*Monopterus albus*) fed high-carbohydrate diets (mean ± S.E.).

	CON	HC	HCF1	HCF2	HCF3	*p*-Value
AST (U/L)	3.45 ± 0.09 ^d^	7.86 ± 0.26 ^a^	3.68 ± 0.04 ^d^	5.47 ± 0.23 ^b^	4.90 ± 0.10 ^c^	<0.001
ALT (U/L)	3.75 ± 0.07 ^c^	9.18 ± 0.41 ^a^	4.21 ± 0.28 ^bc^	4.44 ± 0.2 ^bc^	4.99 ± 0.31 ^b^	<0.001
GLU (mg/dL)	38.72 ± 1.31 ^c^	51.96 ± 1.88 ^a^	42.11 ± 1.74 ^bc^	43.71 ± 0.91 ^b^	40.52 ± 0.80 ^bc^	<0.001
TG (mmol/L)	0.40 ± 0.01 ^b^	0.56 ± 0.01 ^a^	0.40 ± 0.00 ^b^	0.39 ± 0.00 ^b^	0.41 ± 0.01 ^b^	<0.001
TC (mmol/L)	2.72 ± 0.03 ^b^	3.16 ± 0.07 ^a^	2.34 ± 0.04 ^c^	2.44 ± 0.08 ^c^	2.36 ± 0.09 ^c^	<0.001
LDL-C (mmol/L)	3.22 ± 0.03 ^b^	6.30 ± 0.42 ^a^	3.31 ± 0.27 ^b^	2.74 ± 0.27 ^b^	2.86 ± 0.16 ^b^	<0.001
HDL-C (mmol/L)	3.00 ± 0.01 ^b^	3.08 ± 0.05 ^b^	3.40 ± 0.05 ^b^	4.49 ± 0.09 ^a^	4.28±0.31 ^a^	<0.001

Note: In the same row, values with different small letter superscripts mean significant difference (*p* < 0.05). Aspartate aminotransferase (AST), alanine aminotransferase (ALT), glucose (GLU), triacylglycerol (TG), total cholesterol (TC), low-density lipoprotein cholesterol (LDL-C), high-density lipoprotein cholesterol (HDL-C).

## Data Availability

All data generated or analyzed during this study are included in this published article.

## Abbreviations

WGR: weight-gain rate; SR: survival rate; FCR: feed-conversion rate; HSI: hepatosomatic index; VSI: viserosomatic index; CF: condition factor; AST: aspartate aminotransferase; ALT: alanine aminotransferase; GLU: glucose; TG: triacylglycerol; TC: total cholesterol; LDL-C: low-density lipoprotein cholesterol; HDL-C: high-density lipoprotein cholesterol; ROS: reactive oxygen species; PK: pyruvate kinase; GK: glucokinase; G-6-Pase: glucose-6-phosphatase; PEPCK: phosphoenolpyruvate carboxykinase; FBPase: fructose-1.6-bisphosphatase; PFK: phosphofructokinase; CAT: catalase; GSH: glutathione; SOD: superoxide dismutase; GPx: glutathione peroxidase.

## References

[1] FAO (2018). The State of World Fisheries and Aquaculture (SOFIA).

[2] Hatlen B., Grisdale-Helland B., Helland S.J. (2005). Growth, feed utilization and body composition in two size groups of Atlantic halibut (*Hippoglossus hippoglossus*) fed diets differing in protein and carbohydrate content. Aquaculture.

[3] Asaduzzaman M., Wahab M., Verdegem M., Adhikary R., Rahman S., Azim M., Verreth J.A.J. (2010). Effects of carbohydrate source for maintaining a high C: N ratio and fish driven re-suspension on pond ecology and production in periphyton-based freshwater prawn culture systems. Aquaculture.

[4] Stone D.A.J. (2003). Dietary carbohydrate utilization by fish. Rev. Fish. Sci..

[5] Kamalam B.S., Medale F., Panserat S. (2017). Utilisation of dietary carbohydrates in farmed fishe: New insights on influencing factors, biological limitations and future strategies. Aquaculture.

[6] Zhang Q., Chen Y., Xu W., Zhang Y. (2021). Effects of dietary carbohydrate level on growth performance, innate immunity, antioxidant ability and hypoxia resistant of brook trout *Salvelinus fontinalis*. Aquac. Nutr..

[7] Ren M., Ai Q., Mai K., Ma H., Wang X. (2011). Effect of dietary carbohydrate level on growth performance, body composition, apparent digestibility coefficient and digestive enzyme activities of juvenile cobia, *Rachycentron canadum* L.. Aquacult. Res..

[8] Zhang Y., Xie S., Wei H., Zheng L., Liu Z., Fang H., Xie J., Liao S., Tian L., Liu H. (2020). High dietary starch impaired growth performance, liver histology and hepatic glucose metabolism of juvenile largemouth bass, *Micropterus salmoides*. Aquac. Nutr..

[9] Liu D., Zhang Y., Pan M., Yang M., Li X., Fu Y., Gao W., Zhang W., Mai K. (2021). Interactive effects of dietary biotin and carbohydrate on growth performance and glucose metabolism in juvenile turbot *Scophthalmus maximus* L.. Aquaculture.

[10] Liu Y., Liu N., Wang A., Chen N., Li S. (2022). Resveratrol inclusion alleviated high-dietary-carbohydrate-induced glycogen deposition and immune response of largemouth bass, *Micropterus salmoides*. Br. J. Nutr..

[11] Ma H.J., Mou M.M., Pu D.C., Lin S.M., Chen Y.J., Luo L. (2019). Effect of dietary starch level on growth, metabolism enzyme and oxidative status of juvenile largemouth bass, *Micropterus salmoides*. Aquaculture.

[12] Zhang Y., Liang X.F., He S., Chen X., Wang J., Li J., Zhu Q., Zhang Z., Li L., Alam M.S. (2020). Effects of high carbohydrate diet-modulated microbiota on gut health in Chinese Perch. Front. Microbiol..

[13] Lim S.H., Choi C.I. (2019). Pharmacological properties of *Morusnigra* L. (Black Mulberry) as a promising nutraceutical resource. Nutrients.

[14] Wen P., Hu T., Linhardt R.J., Liao S., Wu H., Zou Y. (2019). Mulberry: A review of bioactive compounds and advanced processing technology. Trends. Food. Sci. Technol..

[15] Sarkhel S. (2020). Nutrition importance and health benefits of mulberry leaf extract: A review. J. Pharmacog. Phytochem..

[16] Peng C.H., Lin H.T., Chung D.J., Huang C.N., Wang C.J. (2018). Mulberry Leaf extracts prevent obesity-induced NAFLD with regulating adipocytokines, inflammation and oxidative stress. J. Food Drug Anal..

[17] Zhao X., Li L., Luo Q., Ye M., Luo G., Kuang Z. (2015). Effects of mulberry (*Morusalba* L.) leaf polysaccharides on growth performance, diarrhea, blood parameters, and gut microbiota of early-weanling pigs. Livest. Sci..

[18] Fan L., Peng Y., Wu D., Hu J., Shi X., Yang G., Li X. (2020). Dietary supplementation of *Morus nigra* L. leaves decrease fat mass partially through elevating leptin-stimulated lipolysis in pig model. J. Ethnopharmacol..

[19] Simol C.F., Tuen A.A., Khan H.H.A., Chubo J.K., King P.J.H., Ong K.H. (2012). Performance of chicken broilers fed with diets substituted with mulberry leaf powder. Afr. J. Biotechnol..

[20] Zhu W., Xiao S., Chen S., Xu Q., Yang Z., Liu J., Lan S. (2021). Effects of fermented mulberry leaves on growth, serum antioxidant capacity, digestive enzyme activities and microbial compositions of the intestine in crucian (*Carassius carassius*). Aquac. Res..

[21] Xv Z.C., He G.L., Wang X., Shun H., Chen Y.J., Lin S.M. (2021). Mulberry leaf powder ameliorate high starch-induced hepatic oxidative stress and inflammation in fish model. Anim. Feed Sci. Technol..

[22] Meng Q., Qi X., Fu Y., Chen Q., Cheng P., Yu X., Sun X., Wu J., Li W., Zhang Q. (2020). Flavonoids extracted from mulberry (*Morus alba* L.) leaf improve skeletal muscle mitochondrial function by activating AMPK in type 2 diabetes. J. Ethnopharmacol..

[23] Zhang L., Su S., Zhu Y., Guo J., Guo S., Qian D., Ouyuan Z., Duan J. (2019). Mulberry leaf active components alleviate type 2 diabetes and its liver and kidney injury in db/db mice through insulin receptor and TGF-β/Smads signaling pathway. Biomed. Pharmacother..

[24] Zhong Y., Song B., Zheng C., Zhang S., Yan Z., Tang Z., Kang X., Duan Y., Li F. (2020). Flavonoids from mulberry leaves alleviate lipid dysmetabolism in high fat diet-fed mice: Involvement of gut microbiota. Microorganisms.

[25] Yang J., Chen B., Huang Y., Cao J., Wang G., Sun Y., Chen X. (2017). Effects of dietary mulberry leaf flavonoids on growth performance, body composition, antioxidant indices and resistance to nitrite exposure of genetic improvement of farmed Tilapia (*Oreochromis niloticus*). Chin. J. Anim. Nutr..

[26] Wang Y., Chen B., Cao J., Huang Y., Wang G., Peng K. (2020). Effects of mulberry leaf flavonoids on intestinal mucosal morphology and intestinal flora of *Litopenaeus vannamei*. Chin. J. Anim. Nutr..

[27] Yang D., Chen F., Li D., Liu B. (2000). Requirements of nutrients and optimum energy protein ratio in the diet for *Monoterus albus*. J. Fish. China.

[28] Shi Y., Zhong L., Ma X., Liu Y., Tang T., Hu Y. (2019). Effect of replacing fishmeal with stickwater hydrolysate on the growth, serum biochemical indexes, immune indexes, intestinal histology and microbiota of rice field eel (*Monopterus albus*). Aquac. Rep..

[29] Shi Y., Zhong L., Zhong H., Zhang J., Che C., Fu G., Hu Y., Mai K. (2022). Taurine supplements in high-fat diets improve survival of juvenile *Monopterus albus* by reducing lipid deposition and intestinal damage. Aquaculture.

[30] Shi Y., Zhong L., Zhong H., Zhang J., Liu X., Peng M., Fu G., Hu Y. (2022). Taurine supplements in high-carbohydrate diets increase growth performance of *Monopterus albus* by improving carbohydrate and lipid metabolism, reducing liver damage, and regulating intestinal microbiota. Aquaculture.

[31] Shi Y., Zhong L., Liu Y.L., Zhang J.Z., Lv Z., Li Y., Hu Y. (2020). Effects of dietary andrographolide levels on growth performance, antioxidant capacity, intestinal immune function and microbioma of rice field eel (*Monopterus albus*). Animals.

[32] Livak K.J., Schmittgen T.D. (2001). Schmittgen, Analysis of relative gene expression data using Real-Time Quantitative PCR and the 2^−^^△△CT^ Method. Methods.

[33] Hu Q., Guo W., Gao Y., Tang R., Li D. (2014). Reference gene selection for real-time RT-PCR normalization in rice field eel (*Monopterus albus*) during gonad development. Fish Physiol. Biochem..

[34] Callahan B.J., Mcmurdie P.J., Rosen M.J., Han A.W., Johnson A.J., Holmes S.P. (2016). Dada2: High-resolution sample inference from illumina amplicon data. Nat. Methods.

[35] Susanto A., Hutabarat J., Anggoro S. (2020). The effects of dietary carbohydrate level on the growth performance, body composition and feed utilization of juvenile kelabau (*Osteochilus melanopleurus*). Aquac. Aquar. Conserv. Legis..

[36] Peng K., Wang G., Zhao H., Wang Y., Mo W., Wu H., Huang Y. (2020). Effect of high level of carbohydrate and supplementation of condensed tannins on growth performance, serum metabolites, antioxidant and immune response, and hepatic glycometabolism gene expression of *Lateolabrax japonicus*. Aquac. Rep..

[37] Li S., Li Z., Zhang J., Sang C., Chen N. (2019). The impacts of dietary carbohydrate levels on growth performance, feed utilization, glycogen accumulation and hepatic glucose metabolism in hybrid grouper (*Epinephelus fuscoguttatus* ♀ × *E. lanceolatus* ♂). Aquaculture.

[38] Lin S.M., Shi C.M., Mu M.M., Chen Y.J., Luo L. (2018). Effect of high dietary starch levels on growth, hepatic glucose metabolism, oxidative status and immune response of juvenile largemouth bass, *Micropterus salmoides*. Fish Shellfish Immunol..

[39] Qi S., Wang T., Chen R., Wang C., Ao C. (2017). Effects of flavonoids from *Allium mongolicum Regel* on growth performance and growthrelated hormones in meat sheep. Anim. Nutr..

[40] Li M., Zhu X., Tian J., Liu M., Wang G. (2019). Dietary flavonoids from *Allium mongolicum Regel* promotes growth, improves immune, antioxidant status, immune-related signaling molecules and disease resistance in juvenile northern snakehead fish (*Channa argus*). Aquaculture.

[41] Xu R., Li M., Wang T., Zhao Y.W., Shan C.J., Qiao F., Chen L.Q., Zhang W.B., Du Z.Y., Zhang M.L. (2022). *Bacillus amyloliquefaciens* ameliorates high-carbohydrate diet-induced metabolic phenotypes by restoration of intestinal acetate-producing bacteria in Nile Tilapia. Br. J. Nutr..

[42] Enes P., Panserat S., Kaushik S., Oliva-Teles A. (2009). Nutritional regulation of hepatic glucose metabolism in fish. Fish. Physiol. Biochem..

[43] Han M., Cao X., Zhao C., Yang L., Yin N., Shen P., Zhang J., Gao F., Ren Y., Liang D. (2020). Assessment of glycometabolism impairment and glucose variability using flash glucose monitoring system in patients with adrenal diseases. Front. Endocrinol..

[44] Kim J.Y., Ok H.M., Kim J., Park S.W., Kwon S.W., Kwon O. (2015). Mulberry leaf extract improves postprandial glucose response in prediabetic subjects: A randomized, double-blind placebo-controlled trial. J. Med. Food..

[45] Ren C., Zhang Y., Cui W., Lu G., Wang Y., Gao H., Huang L., Mu Z. (2015). A polysaccharide extract of mulberry leaf ameliorates hepatic glucose metabolism and insulin signaling in rats with type 2 diabetes induced by high fat-diet and streptozotocin. Int. J. Biol. Macromol..

[46] Cross D.A., Alessi D.R., Cohen P., Andjelkovich M., Hemmings B.A. (1995). Inhibition of glycogen synthase kinase-3 by insulin mediated by protein kinase B. Nature.

[47] Zhou C.P., Ge X.P., Liu B., Xie J., Miao L.H. (2013). Effect of high dietary carbohydrate on the growth performance and physiological responses of juvenile Wuchang bream, *Megalobrama amblycephala*. Asian Australas. J. Anim. Sci..

[48] Xu X., Chen N., Liu Z., Gou S., Yin J. (2016). Effects of dietary starch sources and levels on liver histology in largemouth bass, *Micropterus salmoides*. J. Shanghai Ocean Univ..

[49] Dinu D., Marinescu D., Munteanu M.C., Staicu A.C., Costache M., Dinischiotu A. (2010). Modulatory effects of deltamethrin on antioxidant defense mechanisms and lipid peroxidation in *Carassius auratus gibelio* liver and intestine. Arch. Environ. Contam. Toxicol..

[50] Ramalingam M., Kim S.J. (2012). Reactive oxygen/nitrogen species and their functional correlations in neurodegenerative diseases. J. Neural Transm..

[51] Sies H. (1991). Oxidative stress: From basic research to clinical application. Am. J. Med..

[52] Mehra L., Hasija Y., Mittal G. (2016). Therapeutic potential of alpha-ketoglutarate against acetaminophen-induced hepatotoxicity in rats. J. Pharm. Bioallied Sci..

[53] Rymuszka A., Adaszek Ł. (2012). Pro- and anti-inflammatory cytokine expression in carp blood and head kidney leukocytes exposed to cyanotoxin stress-an in vitro study. Fish Shellfish Immunol..

[54] Cao G., Sofic E., Prior R.L. (1997). Antioxidant and prooxidant behavior of flavonoids: Structure-activity relationships. Free Radic. Biol. Med..

[55] Liskova A., Samec M., Koklesova L., Samuel S.M., Zhai K., Al-Ishaq R.K., Abotaleb M., Nosal V., Kajo K., Ashrafizadeh M. (2021). Flavonoids against the SARS-CoV-2 induced inflammatory storm. Biomed. Pharmacother..

[56] Zhao L., Yuan B.D., Zhao J.L., Jiang N., Zhang A.Z., Wang G.Q., Li M.Y. (2020). Amelioration of hexavalent chromium-induced bioaccumulation, oxidative stress, tight junction proteins and immune-related signaling factors by *Allium mongolicum Regel* flavonoids in *Ctenopharyngodon idella*. Fish Shellfish Immunol..

[57] Li M.Y., Guo W.Q., Guo G.L., Zhu X.M., Niu X.T., Shan X.F., Tian J.X., Wang G.Q., Zhang D.M. (2019). Effect of sub-chronic exposure to selenium and Allium mongolicum Regel flavonoids on *Channa argus*: Bioaccumulation, oxidative stress, immune responses and immune-related signaling molecules. Fish Shellfish Immunol..

[58] Banjarnahor S.D.S., Artanti N. (2015). Antioxidant properties of flavonoids. Med. J. Indones..

[59] Shi Y., Hu Y., Wang Z., Zhou J., Zhang J., Zhong H., Fu G., Zhong L. (2021). The protective effect of taurine on oxidized fish-oil-induced liver oxidative stress and intestinal barrier-function impairment in juvenile *Ictalurus punctatus*. Antioxidants.

[60] Xu C., Liu W.B., Remø S.C., Wang B.K., Shi H.J., Zhang L., Liu J.D., Li X.F. (2019). Feeding restriction alleviates high carbohydrate diet-induced oxidative stress and inflammation of Megalobrama amblycephala by activating the AMPK-SIRT1 pathway. Fish Shellfish Immunol..

[61] Jung K.A., Kwak M.K. (2010). The Nrf2 system as a potential target for the development of indirect antioxidants. Molecules.

[62] Baraka S.M., Saleh D.O., Ghaly N.S., Melek F.R., El Din A.A.G., Khalil W.K.B., Said M.M., Medhat A.M. (2020). Flavonoids from Barnebydendron riedelii leaf extract mitigate thioacetamide-induced hepatic encephalopathy in rats: The interplay of NF-κB/IL-6 and Nrf2/HO-1 signaling pathways. Bioorg. Chem..

[63] Goya L., Sarriá B., Ramos S., Mateos R., Bravo L. (2016). Effect of cocoa and its flavonoids on biomarkers of inflammation: Studies of cell culture, animals and humans. Nutrients.

[64] Fan Y., Pedersen O. (2021). Gut microbiota in human metabolic health and disease. Nat. Rev. Microbiol..

[65] Wastyk H.C., Fragiadakis G.K., Perelman D., Dahan D., Merrill B.D., Yu F.B., Topf M., Gonzalez C.G., Treuren W.V., Han S. (2021). Gut-microbiota-targeted diets modulate human immune status. Cell.

[66] Hamer H.M., Jonkers D., Venema K., Vanhoutvin S., Troost F., Brummer R. (2008). The role of butyrate on colonic function. Aliment. Pharmcol. Ther..

[67] Hong J., Jia Y., Pan S., Jia L., Li H., Han Z., Cai D., Zhan R. (2016). Butyrate alleviates high fat diet-induced obesity through activation of adiponectin-mediated pathway and stimulation of mitochondrial function in the skeletal muscle of mice. Oncotarget.

[68] Aalamifar H., Soltanian S., Vazirzadeh A., Akhlaghi M., Morshedi V., Gholamhosseini A., Torfi Mozanzadeh M. (2020). Dietary butyric acid improved growth, digestive enzyme activities and humoral immune parameters in Barramundi (*Lates calcarifer*). Aquac. Nutr..

[69] Teuber M. (1995). The genus Lactococcus. The Genera of Lactic Acid Bacteria.

[70] Vendrell D., Balcázar J.L., Ruiz-Zarzuela I., de Blas I., Gironés O., Múzquiz J.L. (2006). Lactococcus garvieae in fish: A review. Comp. Immunol. Microbiol. Infect. Dis..

[71] Hage-Hülsmann J., Klaus O., Linke K., Troost K., Gora L., Hilgers F., Wirtz A., Santiago-Schübel B., Loeschcke A., Karl-Erich Jaeger K.E. (2021). Production of C20, C30 and C40 terpenes in the engineered phototrophic bacterium Rhodobacter capsulatus. J. Biotechnol..

[72] Murota K., Nakamura Y., Uehara M. (2018). Flavonoid metabolism: The interaction of metabolites and gut microbiota. Biosci. Biotechnol. Biochem..

[73] Song D., Ho C.T., Zhang X., Wu Z., Cao J. (2020). Modulatory effect of Cyclocarya paliurus flavonoids on the intestinal microbiota and liver clock genes of circadian rhythm disorder mice model. Food Res. Int..

[74] Bi Y., Yang C., Diao Q., Tu Y. (2017). Effects of dietary supplementation with two alternatives to antibiotics on intestinal microbiota of preweaned calves challenged with *Escherichia coli* K99. Sci. Rep..

